# Personalized medicine to implementation science: Thiopurines set for the leap

**DOI:** 10.1002/jgh3.12829

**Published:** 2022-10-17

**Authors:** Vishal Sharma, Saurabh Kedia, Vineet Ahuja

**Affiliations:** ^1^ Department of Gastroenterology Postgraduate Institute of Medical Education and Research Chandigarh India; ^2^ Department of Gastroenterology and Human Nutrition All India Institute of Medical Sciences Delhi India

**See article in *JGH Open*, this issue. DOI:** https://doi.org/10.1002/jgh3.12798.

The concept of the four Rs: right drug at the right dose at the right time for the right person is so supremely divine that it borders on being Don Quixotic. Yet, constant inroads by “omics” is bringing into easy grasp a hitherto remote dream. While fully cognizant of the fact that inflammatory bowel disease (IBD) is a heterogeneous disease, the treatment adage “one size fits all” still persists largely because of compulsion of lack of options. Fully knowing that the bewildering heterogeneity of disease hinders kind results for everyone, universal algorithms are still adhered to. Even the most discerning physician with in‐depth knowledge of the nuances of therapeutic tools knows that responses may not go along with expectations. This fjord has been planked by our gradual advances and the promise of precision medicine. Personalized medicine is an emerging practice that uses an individual's genetic profile to guide decisions regarding the prevention, diagnosis, and treatment.^1^

Thiopurines have been gradually falling from grace possibly due to sequential introduction of various targeted monoclonal antibodies. The unbridled enthusiasm to achieve faster and higher treatment goals has led to impatience with the slow‐acting thiopurines in the Western world, where easy access to biologics is not an issue. Recent AGA guidelines appear to bypass thiopurines in their quest for more efficient therapy.^2^ This is despite the constant fact that thiopurines remain a trusted ally of most IBD patients with limited resources. Thiopurines have faithfully served a wide proportion of IBD patients with moderate to excellent results. Certainly, use of thiopurines needs reinvention, and so it is fitting that this drug class is at the forefront of personalized medicine, ahead of other targeted therapies.

## Thiopurine metabolism

**Figure 1:**
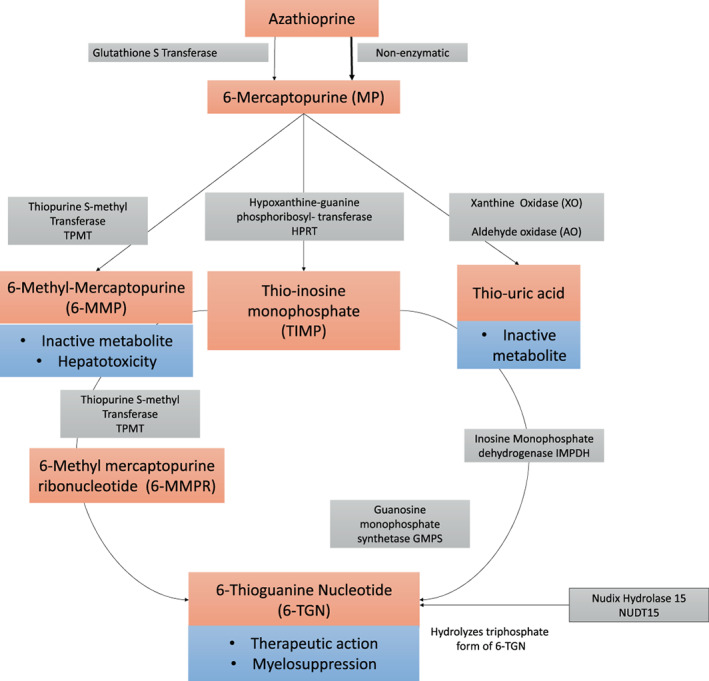
Flowchart showing the important metabolic steps in the metabolism of thiopurines.

Three different thiopurine formulations are clinically useful: azathioprine, 6‐mercaptopurine (6‐MP), and thioguanine (TG). Azathioprine, a pro‐drug, is converted to 6‐MP, enzymatically (glutathione S transferase) as well as non‐enzymatically. As demonstrated in Figure 1, 6‐MP can be channelized through three different pathways, the end products of which impact the efficacy as well as the toxicity of thiopurines.^3^, ^4^ The thiopurine S‐methyl transferase (TPMT) enzyme converts 6‐MP to 6‐methyl mercaptopurine (6‐MMP) and xanthine oxidase converts 6‐MP to thiouric acid, both inactive metabolites. Hypoxanthine‐guanine phosphoribosyl‐transferase (HPRT) enzyme, through a series of enzymatic reactions, converts 6‐MP into active 6‐thioguanine nucleotides (6‐TGN) metabolites (6‐thioguanine triphopshate: 6‐TGTP and deoxy‐6‐thioguanine triphopshate: d′6‐TGTP), which are responsible for efficacy and myelosuppression. 6‐thioguanine, the third therapeutic formulation, bypasses the pathways mentioned above and can restore therapeutic efficacy in patients with ineffective metabolism.^5^

## Genetic polymorphisms impacting thiopurine metabolism

### Common polymorphisms

Changes in the levels of enzymes responsible for gatekeeping the three major metabolic pathways impact the clinical efficacy and adverse effects of thiopurines (Fig. 1). The deficiency or reduction in TPMT levels due to various genetic polymorphisms is well recognized as a cause of thiopurine‐related leukopenia. The pretreatment testing for susceptibility to TPMT‐related leukopenia can be performed either at the phenotypic (testing for TPMT enzyme assay) or the genotypic level (polymorphisms).^6^, ^7^ There are some pros and cons of either test: genotype is not affected by physiological variables, but the testing may miss novel or unrecognized mutations. The phenotypic tests are best performed before starting the therapy because enzyme levels may vary with certain factors (transfusions and age) and decline once thiopurines are initiated. On the other hand, enzyme levels may help detect deficiency even in those where novel mutations are present.^7^ TPMT enzyme assay may misclassify TPMT deficient individuals as TPMT intermediate in a subset.

Although thiopurine‐related leukopenia is more frequent in Asian populations, TPMT mutations are not common. In 2014, a polymorphism in Nudix Hydrolase 15 (NUDT15) was first identified as a cause of thiopurine‐related leukopenia in South Korea.^8^ Subsequently, this polymorphism has been identified as an important cause of thiopurine‐related leukopenia in other Asian countries, including South Asia.^9^, ^10^, ^11^ NUDT15 has a role in hydrolysis of thio‐guanosine‐triphosphate into the monophosphate form. In the absence of NUD15 activity, this conversion is compromised, predisposing such individuals to increased leukopenia with thiopurine use.

### Polymorphisms of unclear significance

Multiple other polymorphisms have been suggested as a possible factor in thiopurine‐related cytopenia, but the evidence is either conflicting or emerging (Table 1).^12^, ^13^, ^14^, ^15^, ^16^, ^17^

**Table 1:** Genes and polymorphisms implicated or investigated for thiopurine‐related cytopenia^12^

Gene	Chromosomal site	Mechanism	Major polymorphisms	Dosing strategy	Comment
Thiopurine methyl‐transferase TPMT	Chromosome 6p22.3	Controls the alternate metabolic pathway to form 6‐methyl mercaptopurine. Deficiency may direct the thiopurine to increased production of thioguanine nucleotides amplifying the toxicity	TPMT*2, TPMT*3A, and TPMT*3C Polymorphisms common in European and American population	Heterozygous: 30–70% of dose Homozygous: Avoid or 10%	Well recognized as a cause of thiopurine‐related cytopenia
Nudix hydrolase or nucleoside diphosphate‐linked moiety X‐type motif 15 NUDT15	13 q14.2	Preferentially hydrolyzes 6‐thio‐guanosine triphosphate to 6‐thio (deoxy)‐guanosine monophosphate Reduced incorporation into DNA and lower cytotoxic and immunosuppressant effects Deficiency increased the triphosphate levels and may increase cytopenias	p.Arg139Cys (R139C) Polymorphisms common in Asians including South Asia	Heterozygous: 30–70% of dose Homozygous: Avoid or 10%	Well recognized as a cause of thiopurine‐related cytopenia in Asians and South Asians
Inosine triphosphate pyrophosphohydrolase ITPase	Chr 20p13	Hydrolyzes ITPA so as to prevent excess 6‐TITP Deficiency will lead to accumulation of 6‐TITP and increased toxicity	rs1127354 or *94 C* > *A* rs7270101 or (*IVS2* + *21 A* > *C*)	NA	May cause neutropenia and other adverse effects like rash, pancreatitis Conflicting evidence
Multidrug‐resistance protein 4 (also known as ABCC4)	Chr 13q32.1	ATP‐dependent efflux of thiopurine metabolites limiting the toxicity Reduced function results in intracellular accumulation of 6‐TGN	rs3765534	NA	Limited evidence
Fat mass and obesity‐associated FTO gene	Chr 16q12.2	Uncertain; Nucleotide demethylase activity of FTO may cleave methyl‐TIMP which is an inhibitor of purine synthesis	rs79206939, p.A134T (susceptibility) rs16952570 (Protective)	NA	Limited evidence, conflicting results
Runt‐related transcription factor 1 RUNX1	Chr 21q22.12	RUNX1 transcription factor has a role in hematopoiesis	(rs2834826, *P* = 5.8 × 10–8)	NA	Limited evidence, Conflicting results

Green: Clinically relevant; pink: Relevance uncertain.

NA, not applicable; 6‐TITP, 6‐thioinosine triphosphate; TPMT, thiopurine S‐methyl transferase.

#### Clinical evidence

The clinical impact of pretherapy genotype testing has been studied in multiple randomized trials. In the TROPIC trial, the pretreatment testing groups (TPMT mutations) had a similar hematological adverse event rate as compared with the standard therapy. Still, the testing was beneficial if only patients with TPMT polymorphisms were considered.^18^ In a multicenter Korean randomized trial, myelosuppression was less frequently noted in patients with polymorphism testing as compared with those who did not (16.7% *vs* 35.9%).^19^ In a Chinese study, where NUDT15 testing was compared with the control group, leukopenia occurred less frequently in the testing arm (23.7% *vs* 32.4%).^20^ Therefore, the evidence through RCTs suggests that the pretherapy genotype assessment reduces the myelotoxicity associated with thiopurine use (Table 2).^18^, ^19^, ^20^

**Table 2:** Randomized control trials which have compared genotype‐based dosing for thiopurines in the setting of inflammatory bowel disease (IBD)^18^, ^19^, ^20^

Reference	Setting	Intervention arm	Control arm	Results
Coenen *et al*.^18^	IBD Multiple centers in Netherlands	Pretherapy screening for 3 TPMT variants‐ homozygous 0‐10% of dose; heterozygous‐50%	Standard therap	Hematologic adverse reactions are similar (7.4% vs 7.9%)
Chang *et al*.^19^	IBD Multiple centers in Korea	Testing for NUDT15, FTO and three common TPMT variants Homozygous: alternative Heterzygous: 50 of azathioprine equivalents	50 mg azathioprine equivalents followed by dose escalation up to 2–2.5 mg/kg	Myelosuppression lower in intervention arm (16.7% *vs* 35.9%)
Chao *et al*.^20^	Crohn's disease Two hospitals in China	NUDT15 C415T CC: standard dose CT: 50% of standard dose, t TT: alternative drugs	Standard dose (2 mg/kg)	Leucopenia lower in intervention arm (23.7% *vs* 32.4%)

ADR, adverse drug reaction; TPMT, thiopurine S‐methyl transferase.

#### Dosing strategies

Because of clear evidence, we usually test for common polymorphisms of TPMT and NUDT15 before initiating thiopurines and modify the dose based on genotype testing.^21^ The presence of a particular genotype should be utilized to classify the phenotype as a normal, intermediate, or poor metabolizer. Poor metabolizers should ideally avoid thiopurines; intermediate metabolizers should be initiated on a reduced dose (30–80%), while normal metabolizers can be started on a normal dose. The pretesting for polymorphisms does not completely obviate the risk of leukopenia completely, and the follow‐up should continue to assess for changes in hemogram and liver function tests.

#### On therapy assessment

##### Metabolite assessment

Once the thiopurines have been initiated, the assessment of metabolites may provide an opportunity to modify the treatment to improve effectiveness and reduce adverse effects. Measurement of two metabolites, as reported by Yeo *et al*. in this issue, could provide an opportunity to address nonresponse and adverse effects of thiopurine therapy.^22^ The measurement of 6‐TGN, which is the active metabolite, assesses the therapeutic benefit of thiopurines. On the contrary, the levels of inactive 6‐MMP estimate the shunting of thiopurines by TPMT. Shunting can cause nonresponse and may need an increase in the dose of thiopurine or the addition of drugs like allopurinol which provide more drug for the HPRT pathway and increase 6‐TGN production. On the contrary, an elevated 6‐TGN in the wake of nonresponse to the thiopurine therapy suggests resistance and warrants a change of therapy. A low level of both 6‐TGN and MMP suggests issues with drug compliance or low dose (Table 3). Although simplistic, the clinical utility of the metabolite measurements has not gained enough currency. The present study adds to the growing evidence and calls for more frequent use of ​​metabolite measurements, especially in difficult cases.

**Table 3:** Use of metabolites in personalizing thiopurine therapy

6‐TGN	6‐MMP	Interpretation and action
235–450 pmol/8 × 10^8^ erythrocytes^†^	High levels if >7500 pmol/8 × 10^8^ erythrocytes^†^	
<235	<5700	Appropriate dose not being administered Check adherence Increase dose
<235	>5700	6‐MMP/TGN > 11 Shunting via the TPMT pathway Add allopurinol
235–450	<5700	Therapeutic dose If there is no clinical effect—consider resistance and change therapy
>450	>5700	Reduce dose If no clinical effect—change to another therapy

^†^ Levels described vary across studies.

6‐MMP, 6‐methyl mercaptopurine; 6‐TGN, 6‐thioguanine; TPMT, thiopurine S‐methyl transferase.

However, there are practical difficulties in using this strategy—the metabolite measurements may not be routinely available, additional costs to a cheap IBD therapy and the levels representing low or high values are not standardized and vary across studies. Further, the data regarding the predictive value of these levels are not homogeneous. In a study comparing two different target levels (600–1200 pmol/8 × 10^8^ RBC *vs* 320–630 pmol/8 × 10^8^ RBC), the higher target levels were associated with numerically more (but not statistically more) discontinuations related to adverse events.^23^ In an RCT, an adjustive strategy to target a TGN level of 250–400 pmol/8 × 10^8^ erythrocytes in Crohn's disease did not achieve a better clinical efficacy.^24^ Observational studies, however, demonstrate conflicting evidence—the mean TGN levels in a study were higher in patients with remission than those who relapsed with ongoing 6‐MP therapy.^25^ Other studies have suggested that a level above the threshold does not guarantee clinical efficacy.^26^ A systematic review suggests that the patients in remission had higher TGN levels than nonresponders and that a level higher than the thresholds (variable 200–260 pmol/8 × 10^8^ RBCs) was associated with increased odds of achieving remission.^27^ Overall, the bulk of evidence suggests that TGN measurement could help in tailoring the dose of thiopurines to improve efficacy and reduce adverse effects.

#### Therapeutic modifications

As suggested by the authors, metabolite assessment and subsequent dose adjustments improved the efficacy and helped 67.2% of patients achieve therapeutic levels after 1 year. About 90% of patients achieved steroid‐free remission, and a similar proportion did not require therapy escalation or surgery. Of 28 patients with subtherapeutic levels, seven were non‐adherent. Dose escalation was done in 17 of 28 patients (including two non‐adherent patients); however, therapeutic 6‐TGN levels were achieved in 5 of 17 patients only, five patients had delayed shunting, and four did not have repeat measurements.^22^ This indicates that dose escalation alone may not circumvent pharmacokinetic non‐response, and other strategies might be required. These include measures that can selectively sway the metabolism toward increased 6‐TGN production. Low‐dose azathioprine along with allopurinol is one such measure (allopurinol is a xanthine oxidase inhibitor that “deshunts” the metabolism towards the HPRT pathway, and metabolites such as thioxanthine inhibit TPMT) that the authors used in shunters and achieved therapeutic 6‐TG levels in ~40% patients. This combination has also been tested in thiopurine‐naïve patients in RCTs and retrospective cohort studies, and though heterogeneous, the evidence suggests higher rates of clinical remission in the combination *versus* azathioprine alone group.^28^, ^29^, ^30^ Although allopurinol–azathioprine combination is an option in shunters, it may also be used at centers where TDM is unavailable. Splitting the dose of azathioprine has also been suggested to overcome thiopurine resistance by reducing 6‐MMP levels via suboptimal substrate affinity for TMPT (and hence reduced TPMT activity) due to reduced dose in each administration. However, the evidence for this approach is minimal and a small pediatric study demonstrated reduced effectiveness as compared with the combination approach.^4^, ^31^

TG bypasses the metabolic pathways utilized by azathioprine/6‐MP and gets directly converted into active 6‐TGNs by HPRT. Hence, there is limited influence by TPMT polymorphisms and lesser production of toxic 6‐MMP and other side metabolites. Therefore, TG is an option in shunters and in those intolerant to azathioprine/6‐MP (except myelosuppression). Pancreatitis is one such indication where TG can be used as previous thiopurine‐induced pancreatitis does not recur with TG. Thioguanine is used in a dose of 20 mg/day (0.2–0.3 mg/kg/day).^5^ Once a feared complication of 6‐TG use, nodular regenerative hyperplasia is not associated with the lower dose used for IBD. Regarding efficacy, in a systematic review, TG treatment after AZA/6‐MP failure showed clinical improvement in 65% of patients.^32^ In a recent study, TG performed better than methotrexate in terms of tolerability and drug survival in patients with Crohn's disease who had failure on conventional thiopurines.^33^

An interesting study highlighted the role of gut microbiome as an important player in thiopurine metabolism and demonstrated their ability to convert TG into active metabolites, and intrarectal release of TG resulted in decreased colitis severity in mouse models.^34^ This was further translated to humans in a recent study on 20 mg thioguanine rectal therapy in the form of enema or suppository. Five of seven patients treated with enemas and eight of nine patients treated with suppositories had clinical improvement along with low systemic 6‐TGN levels.^35^

As shown by the authors and others, though metabolite assessment improved the drug levels and therapeutic efficacy, not all patients responded to adjustment. This could be due to pharmacodynamic failure and lack of perfect concordance between metabolite levels and therapeutic response. The current laboratory techniques measure 6‐TGN and 6‐MMP in the RBCs, which are not the site of action of thiopurines. Measurement in WBCs is, however, technically challenging, which might be overcome in the near future.^4^

#### Handling adverse effects with thiopurines

Another important aspect in prescribing and continuing thiopurines is the assessment and monitoring for adverse events, which can be seen in up to 40% of patients. The authors reported a very low frequency of adverse events, with only 5% of patients developing leukopenia or hepatoxicity. Because of these low numbers, they did not investigate them further.

The adverse events with thiopurines can be dose‐dependent such as myelotoxicity or hepatoxicity and idiosyncratic, such as GI toxicity, pancreatitis, and flu‐like reaction. Long‐term use is associated with systemic immunosuppression‐related adverse effects such as infections and risk of malignancies like lymphoma and nonmelanoma skin cancer (NMSC). Metabolite assessment can help in handling drug‐dependent side effects. Myelosuppression correlates with 6‐TGN levels, while hepatoxicity correlates with 6‐MMP levels. Hence, myelosuppression requires dose reduction or a switch to another agent (depending upon the presence or absence of genetic polymorphisms), and hepatoxicity requires measures for “deshunting” such as allopurinol combination or a switch to TG. Idiosyncratic side effects such as pancreatitis require permanent withdrawal of azathioprine/6‐MP; however, there is some evidence that TG can be used in this situation. Patients having GI upset can be switched to other thiopurine, and evidence suggests that 50% of patients can tolerate the switch.^36^

Long‐term side effects such as malignancy and infections require careful baseline assessment and constant vigilance throughout the therapy. At baseline, screening for hepatitis B and C, and human immunodeficiency virus should be done, and patients should be vaccinated depending upon their immune status and concomitant medications. The risk of Epstein–Barr virus‐associated hepatosplenic T‐cell lymphoma can be mitigated by avoiding thiopurines in young males negative for Epstein‐Barr virus (EBV) serology at baseline. The risk of malignancy may be dependent on the geography and may not be very common in Asians, as reported in a recent retrospective cohort study from northern India, where no case of lymphoma or NMSC was reported over 4788 person‐years of follow‐up.^37^ Hence, thiopurines may be continued if patients tolerate and respond under close monitoring for adverse events, as thiopurine withdrawal is associated with the risk of relapse in 50% patients.^38^

## Conclusion

These forays into precision medicine would remain confined to realms of medical journals unless and until shouldered by implementation science. It is only with implementation science that research results are introduced into routine practice and benefit a large proportion of the population. Implementation science is defined as “which promotes the systematic uptake of research findings and other evidence‐based practices into routine practice, thereby improving the effectiveness of health services.”^39^

The components of implementation science maintain the centrality of local constraints and adapt novel evidence‐based practices and design the implementation practice accordingly. In healthcare settings, the failure to translate what is known to work into the care that patients receive is termed the “know‐do gap.” In simple terms implementation science bridges this know‐do gap. In case of thiopurines, the knowledge gained by personalized medicine evidence is “what works” for IBD patients and implementation science practices should be embraced to leverage “what works” for IBD patients to be disseminated with high efficiency and wide coverage.

For IBD population, the marriage of personalized medicine, evidence‐based innovative practices and their implementation would sustain the use of thiopurines by increasing its efficacy in real‐world setting, which is one great leap that thiopurine prescribers should advocate for.
